# Midkine Attenuates Aβ Fibril Assembly and AmyloidPlaque Formation

**DOI:** 10.21203/rs.3.rs-4361125/v1

**Published:** 2024-06-07

**Authors:** Junmin Peng, Masihuz Zaman, Shu Yang, Ya Huang, Jay Yarbro, Zhen Wang, Danting Liu, Hadeer Soliman, Alex Hemphill, Sarah Harvey, Shondra Pruett-Miller, Valerie Stewart, Ajay Tanwar, Ravi Kalathur, Christy Grace, Martin Turk, Sagar Chittori, Yun Jiao, Zhiping Wu, Anthony High, Xusheng Wang, Geidy Serrano, Thomas Beach, Gang Yu, Yang Yang, Ping-Chung Chen

**Affiliations:** St Jude Children’s Research Hospital; St Jude Children’s Research Hospital; St Jude Children’s Research Hospital; St Jude Children’s Research Hospital; St Jude Children’s Research Hospital; St Jude Children’s Research Hospital; St Jude Children’s Research Hospital; St Jude Children’s Research Hospital; St Jude Children’s Research Hospital; St Jude Children’s Research Hospital; St Jude Children’s Research Hospital; St Jude Children’s Research Hospital; St Jude Children’s Research Hospital; St. Jude Children’s Research Hospital; St. Jude Children’s Research Hospital; St Jude Children’s Research Hospital; St. Jude Children’s Research Hospital; St Jude Children’s Research Hospital; St Jude Children’s Research Hospital; St Jude Children’s Research Hospital; Banner Sun Health Research Institute; Banner Sun Health Research Institute; University of Texas Southwestern Medical Center; Van Andel Institute; St Jude Children’s Research Hospital

## Abstract

Proteomic profiling of Alzheimer’s disease (AD) brains has identified numerous understudied proteins, including midkine (MDK), that are highly upregulated and correlated with Aβ since the early disease stage, but their roles in disease progression are not fully understood. Here we present that MDK attenuates Aβ assembly and influences amyloid formation in the 5xFAD amyloidosis mouse model. MDK protein mitigates fibril formation of both Aβ40 and Aβ42 peptides in Thioflavin T fluorescence assay, circular dichroism, negative stain electron microscopy, and NMR analysis. Knockout of *Mdk*gene in 5xFAD increases amyloid formation and microglial activation. Further comprehensive mass spectrometry-based profiling of whole proteome and aggregated proteome in these mouse models indicates significant accumulation of Aβ and Aβ-correlated proteins, along with microglial components. Thus, our structural and mouse model studies reveal a protective role of MDK in counteracting amyloid pathology in Alzheimer’s disease.

## Introduction

Alzheimer’s disease (AD), an irreversible and progressive neurodegenerative disorder, is the primary cause of dementia^1^. Genetic studies have revealed disease-specific genes (APP, PS1, PS2), high-risk genes (APOE4, TREM2, UNC5C), and a large number of risk loci associated with amyloid, tau, endocytosis, and immunity^2–4^. The dysregulation in the AD proteome within the brain is characterized by the accumulation of Aβ amyloid plaques^5^, tau neurofibrillary tangles^6^, and other aggregated proteins, including TDP-43^7,8^ and U1 snRNP^9–13^. The recent FDA approval of lecanemab^14^, along with the promising Phase III trial results for Donanemab^15^, both being amyloid-targeting antibodies, lend support to the amyloid cascade hypothesis. However, the limited clinical benefits for AD patients suggest the presence of alternative disease mechanisms and imply that intervention at a late disease stage may be insufficient.

Complementary to the genetic search for AD disease genes, we and others have performed large-scale proteomic profiling, uncovering consistent protein alterations in post-mortem AD brains across multiple large cohorts from various independent research groups^16–23^. This has highlighted the deregulation of numerous molecular pathways and protein co-expression modules. Remarkably, a subgroup of proteins is highly upregulated and show a strong correlation with Aβ levels since the early prodromal stage throughout disease progression, including midkine (MDK), pleiotrophin (PTN), netrin 1 (NTN1), netrin 3 (NTN3) and the HTRA1 serine protease^16,19^. Interestingly, these protein changes occur independently of their mRNA transcript levels, indicating post-transcriptional regulation^16,19^. Moreover, these Aβ-correlated proteins have been found in the detergent-insoluble, aggregated proteome, and within amyloid plaques in AD brains, as confirmed by mass spectrometry-based profiling and immunohistochemical analyses^13,24–27^. However, the direct involvement of these Aβ-correlated proteins in plaque formation and AD pathogenesis remains to be elucidated.

The MDK/PTN family of cytokines, consisting of only two members^28^, are among the most closely Aβ-correlated proteins identified in proteomic analyses^16,19^. They are multifunctional heparin-binding factors that promote growth, survival, and migration of various cell types, including neurons and immune cells^28^. In 1993, MDK was reported to localize within the amyloid plaques associated with AD^29^. Preliminary studies suggest that MDK might directly interact with Aβ, displaying high affinity and potentially mitigating Aβ’s toxic effects^30^, but the study had not received attention in the AD research field. Recent proteomic findings support MDK as a top candidate for modulating AD pathogenesis^16,19^, raising interest in fully exploring the role of MDK in AD.

In this study, we have thoroughly characterized the impact of MDK on Aβ assembly in various assays including Thioflavin T, circular dichroism, negative stain electron microscopy (EM), and NMR, demonstrating that MDK protein inhibits the formation of Aβ fibrils. Importantly, the *Mdk* gene knockout (KO) in the background of 5xFAD mice leads to the amyloid increase and microglial activation. Further proteomic profiling of these mouse models reveals the accumulated Aβ and related proteins in the brain of the 5xFAD/KO mice, supporting MDK’s protective role against amyloid pathology in AD.

## Results

### MDK, a top Aβ-correlated protein in human brain, attenuates Aβ fibril assembly

**Figure 1:**
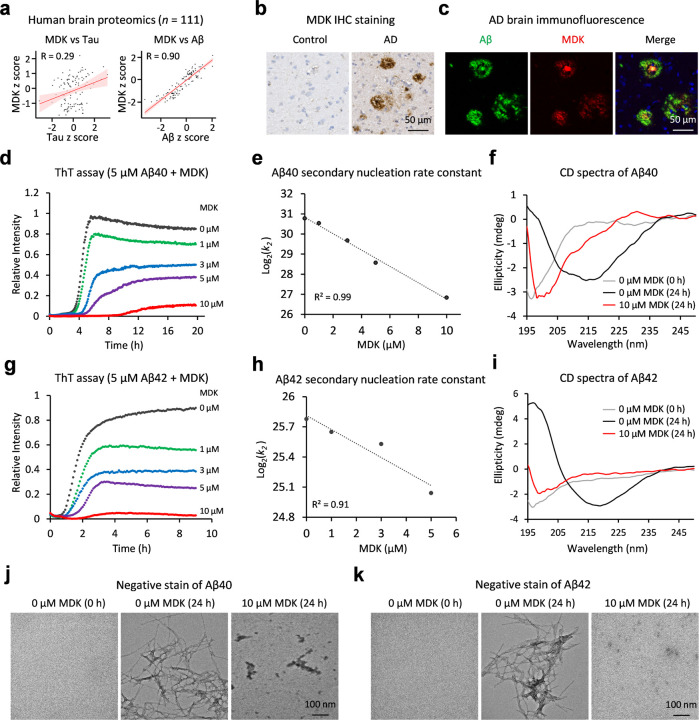
MDK is an Aβ-correlated protein in AD, and it inhibits the fibril assembly of Aβ40 and Aβ42 peptides. **a,** Pearson correlation analysis on MDK in relation to tau or Aβ levels using human brain proteomic datasets (111 cases)^16^. Protein levels were standardized to z scores. **b,** IHC staining of MDK in both control and AD brain tissues, with the scale bar shown. **c,** Co-immunofluorescence staining of Aβ and MDK in AD brain, with the scale bar shown. **d,** The ThT fluorescence assay to detect Aβ40 amyloid fibril formation, with titrated MDK protein (*n* = 3 replicates, averaged data presented). **e,** Analysis of secondary nucleation rate constant (*k*_2_) by globally fitting the ThT data using AmyloFit software^33^, estimating the rate constant of *k*_2_ at each MDK concentration in a unit (M ^−2^ h ^−1^), followed by log transformation and linear fitting of these values at different MDK concentrations. **f,** CD spectroscopy of the samples of Aβ40 mixed with or without MDK, with ellipticity reported in millidegrees (mdeg). **g,** The ThT assay of Aβ42 and MDK (*n* = 3 replicates, averaged data shown). **h,** Analysis of secondary nucleation rate constant of *k*_2_ by AmyloFit. The data points under the condition of 10 µM MDK did not globally fit well with the AmyloFit model, were therefore removed from the fitting. i, CD spectroscopy of the Aβ42/MDK samples. **j-k,** Negative stain EM of Aβ40/MDK and Aβ42/MDK samples, respectively, with the scale bars shown.

Although MDK was identified within the senile plaques of AD patients by antibodies last century^29^, its role in AD has not been well investigated. Recent unbiased proteomic analyses have highlighted MDK as one of the top upregulated proteins highly correlated with Aβ^16,19^. To verify these findings, we re-analyzed an AD proteomic dataset comprising 111 human cases^16^, and performed Pearson correlation analyses of all identified proteins with Aβ and tau levels (Extended Data Table 1). Strikingly, among more than 8,400 identified proteins, MDK shows the best correlation with Aβ levels (R = 0.90) (Fig. 1a). In contrast, MDK displays a much weaker correlation with tau (R = 0.29), consistent with the idea that Aβ and tau do not strongly correlate during the progression of AD (R = 0.26). The presence of MDK in the plaque regions of the human brain was further confirmed through immunohistochemical (IHC) staining and immunofluorescent co-staining with Aβ peptides (Fig. 1b, 1c). Additionally, in the 5xFAD amyloidosis mouse model (also termed FAD for simplicity), which overexpresses mutant human APP and PSEN1 genes^31^, MDK accumulation was observed to be higher in FAD mice than in wild type (WT), corroborated by antibody-based detection of MDK in mouse plaques (Extended Data Fig. 1a, 1b, 1c). These findings not only reaffirm the strong Aβ-MDK correlation in human but also suggest the FAD mice as a valuable model for studying the role of MDK in AD pathogenesis.

Given the previous reports that MDK protein directly binds to Aβ peptides^16,30^, we investigated its impact on Aβ fibrillization using various biophysical assays. Highly purified recombinant human MDK protein (Extended Data Fig. 1d) at concentrations ranging from 1 µM to 10 µM, was incubated with synthetic Aβ40 peptide at 5 µM. The fibrillization process was monitored by the fluorescence of thioflavin T (ThT), in which the fluorescence intensity increases upon binding with amyloid fibrils^32^. As anticipated, in the absence of MDK, Aβ40 displayed a time-dependent increase in fibrillization, with increased lag time and decreased ThT fluorescence intensity in a dose-dependent manner (Fig. 1d). We further turned to the AmyloFit software^33^ to determine effects of MDK on the microscopic steps during the aggregation of Aβ. The best fit revealed that MDK negatively impacts Aβ40 secondary nucleation rate constant (*k*_2_) and elongation constant (*k*_+_), as both log_2_(*k*_2_) and log_2_(*k*_+_) values show a linear decrease with increasing MDK concentrations (Fig. 1e, Extended Data Fig. 1e, 1f). Following a 24 h incubation, far UV circular dichroism (CD) analysis of the Aβ40 samples confirmed the inhibitory effect of MDK on Aβ40 fibrillization. Aβ40 in absence of MDK initiated from unstructured random coil confirmation (0 h) to β-sheet structure (24 h). However, in the presence of MDK (10 µM) Aβ40 essentially retained into the unstructured confirmation like monomers (Fig. 1f). We extended this study to synthetic Aβ42 peptide, yielding similar outcomes (Fig. 1g, 1h, 1i, Extended Data Fig. 1g). Moreover, the Aβ40/Aβ42 samples, with and without MDK, were examined using negative stain EM analysis (Fig. 1j, 1k). Amyloid fibrils were observed as the end-point aggregates of samples of Aβ40/Aβ42 peptide (5 µM) alone. In contrast, the presence of high concentration of MDK (10 µM, 2:1 molar ratio) produced some nonfibrillar aggregates, demonstrating MDK’s potential to inhibit Aβ fibril formation effectively. It is important to note that MDK alone does not fibrillate under similar conditions as confirmed by ThT binding assay, far UV CD, and negative stain results (Extended Data Fig. 1h, 1i, 1j). Together, these results suggest that midkine is inhibiting the formation of amyloid fibrils *in vitro*.

In spite of the inhibition effect of MDK on Aβ assembly, we observed that Aβ could still assemble as fibrils in the presence of lower MDK concentrations, such as a 1:1 molar ratio, and subsequently examined the Aβ42 fibrils under this condition using negative stain and immunogold electron microscopy. Although the resolution was low, the Aβ42 fibrils appeared to be surrounded by protein molecules (Extended Data Fig. 2a). As recombinant Aβ fibrils assemble differently from endogenous Aβ filaments found in human brains^34–38^, we further investigated the presence of MDK in Aβ fibrils extracted from human AD brains. Immunogold negative stain EM with the MDK antibodies revealed that MDK molecules are clustered around these Aβ fibrils (Extended Data Fig. 2b). Moreover, we utilized the AlphaFold2-multimer prediction tool^39^ to hypothesize possible Aβ-MDK interacting interfaces, suggesting that the N-terminus of Aβ (residues 1–12) might interact with the C-terminus of MDK (residues 74–80 and 84–89) (Extended Data Fig. 2c, 2d).

**Figure 2:**
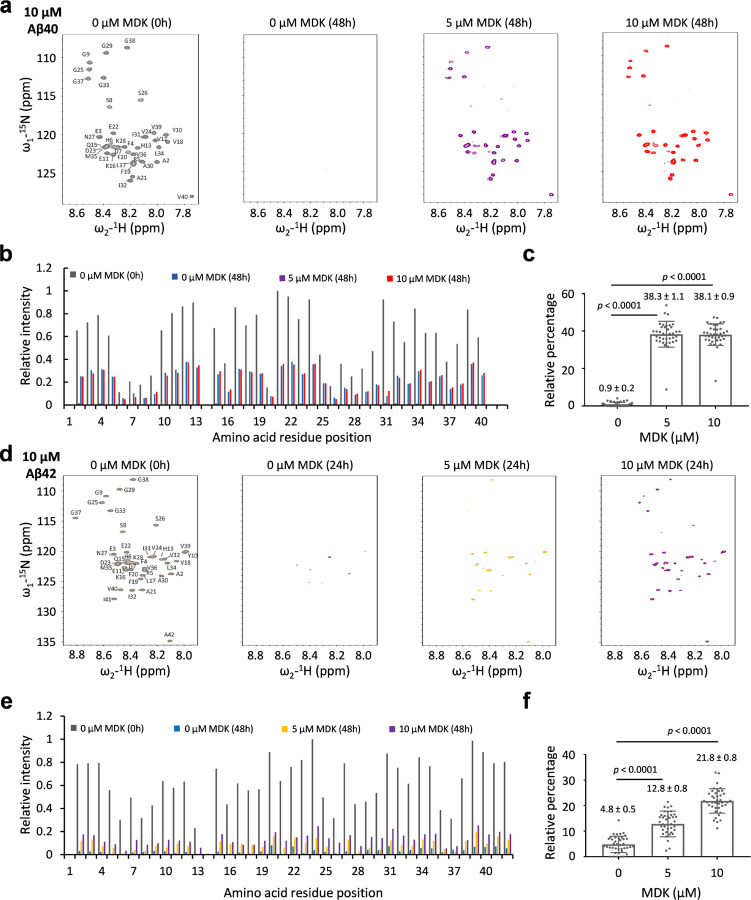
MDK rescues NMR signals of Aβ peptides. **a,** ^1^H-^15^N HSQC spectra of Aβ40 (10 μM) with MDK concentrations of 0, 5, or 10 μM in a buffer containing of 50 mM Tris buffer, pH 7.5. The spectra were collected prior to incubation (the left panel) and after 48 h incubation (the right three panels). **b,** Relative cross peak intensities for each residue, excluding D1 and H14. The intensities were normalized with the maximum intensity set to 1. **c,** Relative percentage of Aβ40 intensities after 48 h incubation compared to those before incubation. The percentage values were calculated for each residue and then averaged to show the mean and standard error of the mean (SEM). Statistical significance is analyzed by two-tailed Student’s *t*-test. **d-f,** The HSQC spectra of Aβ42 under similar conditions as Aβ40, but with the incubation time shortened to 24 h. Statistical significance is analyzed by two-tailed Student’s *t*-test.

To further confirm the effect of MDK on Aβ fibrillization, we acquired ^1^H-^15^N HSQC NMR spectra of ^15^N-labeled monomeric Aβ40 (10 µM) with MDK titration (0, 5, and 10 µM, Fig. 2a). Reference spectra were acquired prior to incubation, with amide cross peaks assigned to specific residues^40^. After 48 h, a notable disappearance of cross peaks suggested Aβ40 peptide fibrillization. Remarkably, with MDK co-incubation at varying concentrations, the cross peaks re-appeared although at reduced intensities. As the HSQC cross peak intensities correlate with monomeric Aβ concentration^41^, we quantified the cross peak intensities for each residue (Fig. 2b), and calculated the averaged percentage relative to the pre-incubation reference of Aβ40. The MDK addition (5 µM and 10 µM) rescued the cross peak intensities from 0.9 ± 0.2% to 38.3 ± 1.1% and 38.1 ± 0.9%, respectively (Fig. 2c). We also observed a similar impact of MDK on Aβ42 fibrillization using a protocol of 24 h incubation (Fig. 2d). However, the same concentrations of MDK were less effective in recovering Aβ42 cross peaks compared to Aβ40 (Fig. 2e, 2f). For example, the MDK addition (5 µM and 10 µM) retrieved the cross peak intensities from 4.8 ± 0.5% to 12.8 ± 0.8% and 21.8 ± 0.8%, respectively. This aligns with our ThT, CD and negative stain EM data, supporting the notion that Aβ42 is more aggregation prone than Aβ40^37^. In summary, our comprehensive biophysical characterization strongly demonstrates that MDK can modulate Aβ fibrillization in vitro.

### Genetic deletion of Mdk gene in FAD mice increases amyloid plaques and microglial activation.

**Figure 3:**
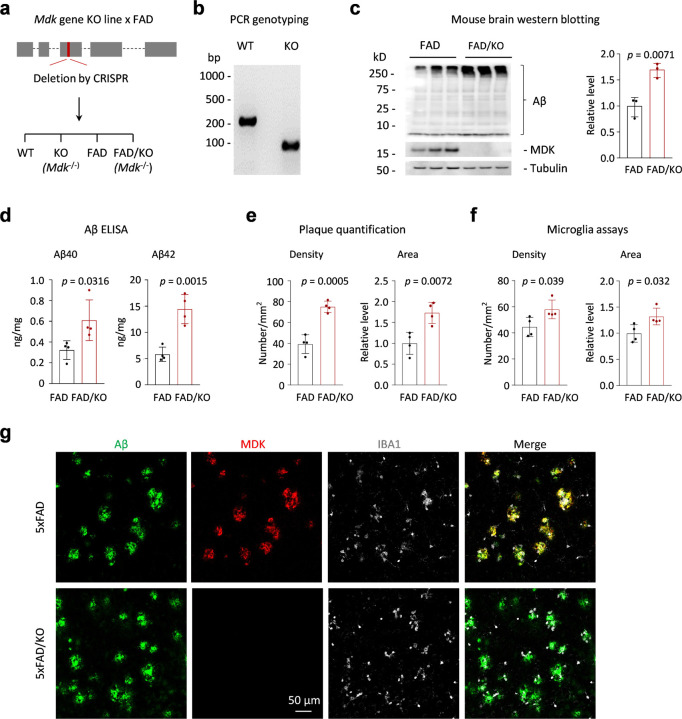
*Mdk* gene knockout in FAD results in Aβ accumulation, plaque increase and microglia activation. **a,** The diagram of the *Mdk* gene structure, which comprises five exons, including the first exon as the 5’ untranslated region. We targeted a CRISPR-mediated deletion of a specific DNA segment within exon 3, resulting in a gene knockout that disrupts the open reading frame sequence. Crossbreeding of the *Mdk* KO with FAD resulted in the generation of four genotypes for comparisons. **b,** PCR genotyping of the WT and homogenous *Mdk* KO. **c,** Western blotting to confirm the loss of MDK protein and the increase of Aβ in the brain of heterogenous FAD mice with homogenous *Mdk* KO (12-month-old, *n* = 3 replicates). **d,** Aβ ELISA analysis (*n* = 4 replicates). **e,** Quantification of amyloid plaque density and area (*n* = 4 replicates). **f.** Quantification of microglia density and area (*n* = 4 replicates). **g.** Co-immunofluorescence staining of Aβ, MDK and IBA1 in the mouse brains. Scale bar, 50 µm. Statistical significance is analyzed by two-tailed Student’s *t*-test.

To investigate the role of MDK in Aβ fibrillization in vivo, we generated a mouse model with an *Mdk* gene knockout by CRISPR-mediated deletion of 23 base pairs in exon 3 of the mouse genome (Fig. 3a). The deletion was confirmed through genome sequencing and PCR analysis (Fig. 3b), with the absence of MDK protein verified by western blot analysis (Fig. 3c). We crossbred this knockout line with the FAD mice to produce offspring that were either FAD mice with or without the *Mdk* knockout. The *Mdk* knockout resulted in a marked increase in Aβ levels in the cortex of 12-month-old mice brains, as shown by western blot analysis (Fig. 3c). Further, ELISA analysis indicated elevated accumulation of both Aβ40 and Aβ42 peptides in the brain lysate (Fig. 3d). These biochemical findings indicate that *Mdk* gene knockout contributes to the increased accumulation of two major Aβ species in the FAD genetic background.

Next, we characterized the FAD/KO mice through immunostaining to evaluate the effects of *Mdk* deletion on plaque formation and microglial activation in the brain. As anticipated, *Mdk* knockout resulted in increased plaque density and area in the cortex of 12-month-old mouse brains (Fig. 3e), which was accompanied by heightened microglial activation, evidenced by the rise in microglia density and area (Fig. 3f). In subsequent immunofluorescence staining studies, amyloid plaques detected by the Aβ antibody were found to highly colocalize with MDK protein in FAD mice, and the MDK staining was absent in the knockout mice (Fig. 3g). Additional co-staining with IBA1, a protein marker for microglia^42,43^, revealed a significant accumulation of microglia around the plaque areas (Fig. 3g), in agreement with the observed increase in microglial density and area in the FAD/KO mice. These results underscore that *Mdk* gene knockout leads to enhanced plaque formation and microglial activation in the FAD mouse model.

**Figure 4:**
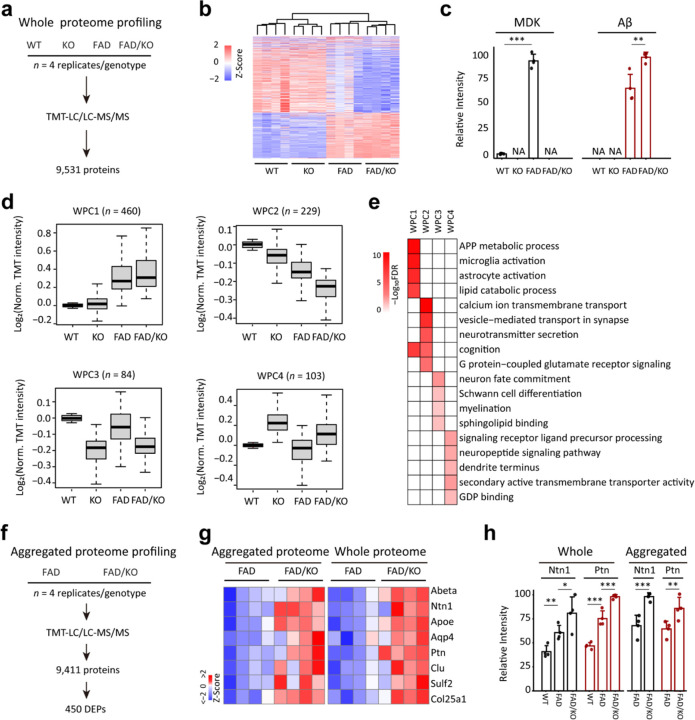
Brain tissue proteomics reveals *Mdk* knockout causes the accumulation of Aβ and Aβ-correlated proteins, along with microglia activation in FAD. **a,** Workflow of whole proteome analysis of the brain cortical region for four mouse genotypes (12-month-old, *n* = 4 replicates for each genotype). **b,** Clustering of the whole proteome to show the grouping of four genotypes using DEPs. **c,** MDK and Aβ levels extracted from the whole proteome analysis. **d,** Four major clusters of DEPs defined by the WGCNA program. The intensity of each protein is log transformed followed by Z score analysis. **e,** Enriched pathways in the WPC proteins by Fisher’s exact test. **f,** Workflow of profiling the aggregated proteome in FAD and FAD/KO mice (12-month-old, *n* = 4 replicates). **g,** Heatmap for selected proteins shared by the whole proteome and aggregated proteome. The data are shown after normalization by z score transformation. **h,** Ntn1 and Ptn protein levels selected from the whole and aggregated proteome analyses. Statistical significance is determined by a two-tailed Student’s *t*-test, and the results are shown as mean ± SEM. *: P < 0.05; **: P < 0.01; and ***: P < 0.001.

To understand the impact of *Mdk* knockout in the FAD mice at the molecular level, we analyzed the cortical brain proteome across four genotypes: WT, KO, FAD, and FAD/KO (Fig. 4a, 4 replicates per genotype). Employing a refined tandem-mass-tag coupled with two-dimensional liquid chromatography and tandem mass spectrometry (TMT-LC/LC-MS/MS) platform^13,44–46^, we identified 113,297 peptides, corresponding to 9,531 proteins (8,365 genes) with false discovery rate (FDR) below 0.01, which were quantified across all these samples (Extended Data Tables 2–3). After correcting sample loading bias, principal component analysis (PCA) and clustering analysis clearly separate distinct mouse groups, indicating the high quality of the proteomic data (Fig. 4b, Extended Data Fig. 3a-3b). As expected, MDK protein levels were elevated in FAD mice compared to WT and reduced to noise levels in knockout mice (Fig. 4c). In FAD mice, *Mdk* deletion led to a significant increase of Aβ peptide levels (Fig. 4c).

To identify the differentially expressed proteins (DEPs) among the four genotypes, we performed an ANOVA analysis using a previously reported method^16^ with two criteria: (i) a P-value of less than 0.05, and (ii) a log2 fold change (FC) greater than twice the standard deviation (2 SD) in any of the three pairwise comparisons: WT vs KO, FAD vs WT, or FAD/KO vs FAD (Extended Data Fig. 3c). Following this analysis, a total of 876 DEPs were accepted (Extended Data Table 4), with a final FDR below 0.1 as determined by permutation analysis^47^. We then classified these DEPs into four whole proteome clusters (WPCs) using the weighted gene co-expression network analysis (WGCNA)^48^, followed by pathway enrichment analysis (Fig. 4d, Extended Data Tables 4–5). In detail, WPC1 (*n* = 460) exhibits a gradual increasing trend across the four genotypes from WT, KO, FAD to FAD/KO. This cluster includes Aβ and Aβ-correlated proteins^16^, such as Ntn1, Ptn, Clu, and Col25a1, as well as some marker proteins for microglia and astrocytes: Trem2, Ctss, Olfml3, Csf1r, Itgb5, Gfap, Aqp4, Pla2g7, Gja1, and Slc1a3^49,50^ (Extended Data Fig. 3d), and it is enriched in four representative pathways: APP metabolic process, microglia activation, astrocyte activation and lipid catabolic process (Fig. 4e, Extended Data Table 6). In contrast, WPC2 (*n* = 229) shows notable decrease along the four genotypes, related to the pathways of calcium ion transmembrane transport, vesicle-mediated transport in synapse, neurotransmitter secretion, cognition, and G protein-coupled glutamate receptor signaling. Interestingly, this cluster includes the AD risk gene/protein Bin1 that regulates calcium homeostasis and neuronal excitability^51,52^.

In addition, both WPC3 (*n* = 84) and WPC4 (*n* = 103) display effects of *Mdk* knockout that are independent of the FAD genetic background. Proteins in WPC3 decrease in *Mdk* KO mice and are enriched in four major pathways: neuron fate commitment, Schwann cell differentiation, myelination, and sphingolipid binding (Fig. 4e, Extended Data Table 6). For instance, the cluster contains Pou3f1 (also known as Oct-6), which is a major downstream effector of cAMP in Schwann cells and a key transcription factor for myelination in the peripheral nervous system^53,54^. WPC4 primarily consists of proteins that are significantly upregulated due to *Mdk* knockout, enriched in the pathways of signaling receptor ligand precursor processing, neuropeptide signaling pathway, dendrite terminus, secondary active transmembrane transporter activity and GDP binding. For example, prohormone convertase 1 (Pcsk1) is a critical endopeptidase required for the processing of neurotransmitters and hormones^55^. Overall, the protein alterations found in WPC3 and WPC4 likely reflect the developmental function of midkine, implicating a pleiotropic impact of *Mdk* knockout. Nevertheless, within WPC1, we observed a distinct increase in Aβ and Aβ-correlated proteins, along with the activation of microglia and astrocytes, supporting that *Mdk* knockout exacerbates pathological severity.

Furthermore, we validated the enhanced pathology in FAD/KO mice by profiling the detergent-insoluble, aggregated proteome in both FAD and FAD/KO mice, identifying 131,400 peptides from 9,411 unique proteins (8,236 genes, Fig. 4f, Extended Data Table 7). After performing quality control analyses, such as normalization and PCA (Extended Data Fig. 3e, 3f), we found that 450 DEPs were elevated in the aggregated proteome of FAD/KO mice compared to FAD mice (Extended Data Fig. 3g, Extended Data Table 7). Remarkably, the FAD/KO mice showed an accumulation of Aβ and other plaque-associated proteins in the aggregated proteome relative to FAD mice. A heatmap highlights some of these proteins, consistently detected in both the whole and aggregated proteomes, including Ntn1, Apoe, Aqp4, Ptn, Clu, Sulf2, and Col25a1^22^ (Fig. 4g, 4h).

In summary, the knockout of *Mdk* gene within the FAD mice resulted in an increase in Aβ and Aβ-associated proteins, amyloid plaque formation, and microglial activation. This was substantiated through experiments of western blotting, ELISA, immunostaining of brain tissues, and extensive proteomic profiling of both the whole and aggregated proteomes in the brain.

## Discussion

Recent proteomics studies of Alzheimer’s disease (AD) brains have identified a core set of proteins, including MDK, PTN, NTN1, NTN3, and SFRP1, that share several characteristics: (i) a high correlation with Aβ throughout AD progression; (ii) a prominent presence in the aggregated proteome; and (iii) enrichment in amyloid plaques^16,19^. However, the role of most of these proteins in AD pathogenesis remains underexplored. We focused on MDK for its potential impact on Aβ fibril assembly in vitro and amyloid plaque development in vivo. Our findings show that MDK disrupts Aβ fibril assembly, and its genetic deletion exacerbates plaque accumulation in the FAD mouse model. Considering the extended prodromal period before clinical AD onset, early Aβ accumulation may activate compensatory mechanisms to mitigate Aβ toxicity. Our research indicates that MDK could be one such factor, offering protective effects against Aβ-induced toxicity and contributing to resistance against amyloid pathology.

MDK protein influences the fibrillation of Aβ40 and Aβ42 peptides, primarily through its inhibitory actions on the elongation of Aβ filaments (*k*_+_ rate constant) and the secondary nucleation phase of fibrillation (*k*_2_ rate constant). This influence is demonstrated by AmyloFit analysis of ThT fluorescence data, an approach previously used to study how inhibitors affect specific microscopic steps during the Aβ aggregation^33,41,56^. Several other proteins, such as the DNAJB6 and Brichos molecular chaperones, have been found to interfere with Aβ fibrillation through primary and secondary nucleation mechanisms^57,58^, whereas monomeric α-synuclein can block the secondary nucleation of the Aβ fibrils rather than primary nucleation^59^. Similar to monomeric α-synuclein, MDK might interact with Aβ oligomers rather than Aβ monomer, suppressing the Aβ fibrillation through inhibiting the secondary nucleation. Investigating the Aβ-MDK interaction at an atomic level by cryogenic electron microscopy in future studies will provide more mechanistic insights and could be important for rational design of effective therapeutic strategies in AD pathogenesis.

While *Mdk* knockout’s impact on amyloid accumulation in the FAD model can be attributed to its inhibitory effect on Aβ assembly, the *Mdk* knockout may have a pleiotropic effect. We observed an upregulation of PTN, another member of the midkine/pleiotrophin family. As PTN might have similar function to MDK, this upregulation could act as a compensatory mechanism mitigating the effects of MDK loss on amyloid formation. In addition, MDK has reported to interact with various membrane receptors, including PTPζ (a transmembrane tyrosine phosphatase)^60^, LRP-1^61^, integrins (α6β1 and α4β1)^62^, Neuroglycan C^63^, ALK (anaplastic lymphoma kinase)^64^, and Notch-2^65^, regulating development, cell migration and inflammation during brain injury^28^. Although the role of most of these MDK receptors in AD remains underexplored, LRP-1 is a receptor of the AD risk factor APOE, which regulates lipid hemostasis in AD development^66^. In our proteome analysis of *Mdk* KO mice, although there were no significant changes in the protein levels of these known MDK receptors, we did observe alterations of a subset of proteins (WPC3 and WPC4) resulting from the knockout of *Mdk* gene, independent of the FAD genetic background. These perturbed proteins might influence amyloid pathology. Nevertheless, our results reveal a potential role of MDK as a resilience factor in the early stage of developing amyloid pathology, offering a promising avenue for therapeutic intervention against the disease’s progression.

## Methods

All experiments performed in this study comply with all relevant ethical regulations. All animal experiments were approved by the Institutional Animal Care and Use Committee (IACUC) at St Jude Children’s Research Hospital.

### Statistics and reproducibility.

Sample sizes were not predetermined using statistical methods; however, they closely resembled those reported in previous publications^68–70^. While the assumption of normal data distribution was made, formal testing for normality was not conducted. Statistical methods were subsequently employed to estimate p-values, and the analysis of false discovery rate was conducted. No exclusions of animals occurred in the study, and the experiments were not randomized. Animals were utilized whenever available without randomization. The investigators were blinded to genotype information during the quantitation of immunostaining for plaque and microglia.

### Human brain samples.

Human postmortem brain tissue samples (frontal gyrus) were provided by the Brain and Body Donation Program at Banner Sun Health Research Institute. Clinical and pathological diagnoses were based on the established criteria^67^.

### Mouse model.

All mice (C57BL/6J) were housed under a 12-h light: 12-h dark cycle at 22–25 °C and 40%-60% humidity. *Mdk* knockoutmice were generated using CRISPR-Cas9 technology to partially delete coding region in the genome to cause frame shift mutation. Briefly, dual gRNAs were designed with *in silico* off-target analysis to determine highly unique spacer sequence with at least 3bp of mismatch to any other site in the mouse genome. In addition, we also consider the targeted region covered among all isoforms, and select CAGE257.MDK.g2 (5’-UGCGGCAUGGGCUUCCGCGA-3’) and g14 (5’-GCACCUUGCAAUGGACGCGC-3’). Precomplexed ribonucleic proteins (RNPs) consisting of 25 ng/µl of each sgRNA (Synthego) and 50 ng/ µl SpCas9 mRNA (Trilink) were injected into the cytoplasm of C57BL/6J fertilized zygotes and transferred to CD-1 psuedopregnant fosters. Resulting animals were genotyped by targeted next generation sequencing using primers CAGE257.F: 5’-GTGAGGCAGGCCGTGTGACCAAGTG-3’ and CAGE257.R:5’-TGCAGTCGGCTGATGGGAGAGTGGC-3’ and analyzed using CRIS.py as previously described^68,69^ Founder animals with out-of-frame mutation were backcrossed to C57BL/6J mice for two generations. A founder carrying a 23bp deletion in exon 2 or 3 of all isoforms was selected for the study. FAD mice (B6.Cg-Tg(APPSwFlLon,PSEN1*M146L*L286V)6799Vas/Mmjax) were obtained from Jackson Laboratory (MMRRC_034848-JAX) and underwent backcrossing with C57BL/6J for 10 generations. *FAD/Mdk*^−/−^ (FAD/KO) mice were generated by crossing with *FAD/Mdk*^+/−^ with *Mdk*^+/−^mice. In all mouse experiments, same ratio of male and female animals was utilized.

### Western blotting.

Protein lysates underwent separation via 4–20% Tris-Glycine gel and transfer to 0.22 µm nitrocellulose membranes. Blots underwent a 1-h blocking with 3% BSA for MDK or 5% skim milk for other targets. The following primary antibodies were incubated overnight at 4°C: anti-mouse MDK (1:1000, sheep polyclonal, R&D, #AF-7769), anti-Aβ (1:1000, mouse monoclonal, clone 82E1, IBL, #10323), and anti-β-tubulin (1:5000, rat monoclonal, clone YOL1/34, abcam, #ab6161). After three 5-minute washes, blots were exposed to secondary antibodies conjugated with HRP (1:40,000, Jackson ImmunoResearch) for 1 h at room temperature, followed by another three 5-minute washes. Chemiluminescence was developed by using either the SuperSignal West Pico PLUS or Femto substrate (Thermo Fisher Scientific) and subsequent detected by ChemiDoc system (Bio-Rad).

### ELISA analysis.

Aβ40 and Aβ42 levels in mouse brain lysate were measured using Meso Scale Diagnostics (MSD) ELISA plate (V-PLEX Aβ Peptide Panel 1 (6E10) Kit)^70^. Briefly, the 96-well plate coated with Aβ40 and Aβ42 antibodies were blocked with blocker diluent for 1 h at room temperature to reduce non-specific protein binding. The plate was washed 3 times with 150 µl /well MSD wash buffer. Then 25 µl of detection antibody followed by 25 µl of diluted standards or mouse brain lysate were loaded and incubated for 2 h. The plate was washed 3 times with 150 µl /well wash buffer. Finally, signals were developed by adding 150 µl of 2x read buffer followed with immediately read on the MESO SECTOR S 600 (MSD).

### Immunohistochemistry.

The staining was modified from previously report^12^. Briefly, brain tissue samples were fixed with 10% formalin buffer, and embedded in paraffin. 5–10 µm sections were deparaffinized, rehydrated and rinsed with water. Antigen retrieval was performed with 10 mM citric buffer (pH 6.0) with boiled water bath for 20 min and cooled down to room temperature. Endogenous peroxidase activity is blocked by 1% H_2_O_2_ in PBS buffer for 10 min. After rinsed by PBS, sections were then blocked with 5% donkey serum in PBS with 0.3% Triton X-100 (PBST) for 30 min at room temperature followed with primary antibodies diluted with PBST plus 2% BSA and 0.2% skim milk for overnight at 4°C: anti-human MDK, (goat polyclonal, 1:200, R&D, #AF-258-SP), anti-mouse MDK (1:200, sheep polyclonal, R&D, #AF-7769), anti-Aβ (1:100, clone 82E1, IBL, #10323), anti-IBA1 (1:400, rabbit polyclonal, Fujifilm Wako, #019-19741). After washing with PBS, sections were then incubated with secondary antibodies (1:500, Jackson ImmunoResearch) for 1 hour at room temperature. For immunofluorescence staining of Aβ and IBA1, we used Alexa Fluor 488 and Cy3-conjugated secondary antibodies, respectively. For MDK, we went with HRP polymer-conjugated secondary antibodies (Vector Laboratories, # MP-7405). Signal was amplified using TSA-Cy5 (Akoya Biosciences). For chromogenic immunodetection, biotin-conjugated secondary antibodies were used followed with ABC reaction by Vectastain ABC kit (Vector Laboratories, #PK-4000) and developed by 3,3-diaminobenzidine solution (Vector Laboratories, #SK-4100). Images were captured by Zeiss Axioscan.Z1 and Zeiss LSM 780 confocal microscopy.

### Quantitation of Aβ plaques and microglia.

Brain tissue serially sectioned sagittally at 10 μm thickness. Three slides, each representing every 30th section, were utilized for immunohistochemistry followed with imaging captured with Zeiss Axioscan.Z1. QuPath, an open-source software^71^ was carried out for quantitation of density and area of Aβ plaques and microglia in an investigator blinded manner.

### MDK protein purification.

The MDK construct (22–143 aa) was over-expressed in BL21 (DE3) Rosetta 2 cells. A single colony was employed to cultivate a starter culture overnight. Subsequently, 10 ml of the starter culture was utilized to inoculate 1L of 2XYT media, which contained 50 μg/ml Kanamycin and 35 μg/ml Chloramphenicol. The inoculation took place at 37°C, with agitation at 220 rpm, until the cell density reached an OD600 nm of approximately 0.8. The induction of protein over-expression was initiated by adding 0.5 mM Isopropyl β-D-thiogalactopyranoside (IPTG), and the culture was further incubated at 18°C for 16 h. The cells were harvested by centrifugation at 5000 rpm for 15 min at 4 °C and the cell pellet was frozen in −80°C until used. The cell pellet was resuspended in lysis buffer (20mM HEPES, pH 7.4, 500mM NaCl, 5% glycerol), supplemented with an EDTA-free complete protease inhibitor cocktail tablet (Roche). Disruption was achieved using a cell disruptor (Constant Systems Ltd.) at 20.0 kPSI pressure in 2 rounds of lysis. The resulting lysate was cleared by centrifugation at 20,000 rpm for 30 min at 4 °C and subsequently applied to a gravity column following a 30-minute incubation with pre-equilibrated Ni-NTA resin at 4 °C. The column was then washed by 10 CV of wash buffer A (20mM HEPES pH 7.4, 500mM NaCl, 12.5 mM Imidazole and 5% glycerol), followed by 10 CV of wash buffer B (20mM HEPES pH 7.4, 500mM NaCl, 50 mM Imidazole and 5% glycerol). The protein was eluted with 20mM HEPES pH 7.4, 500mM NaCl, 250 mM Imidazole and 5% glycerol. The eluted fractions were analyzed by loading onto a 4–20% SDS-PAGE gel. Subsequently, the elution fractions were combined, and the His-tag was cleaved from the protein using tobacco etch virus (TEV) protease overnight at 4 °C. The cleaved protein was then further purified by size exclusion chromatography using a Superdex 75 16/600 column (Cytiva) equilibrated with 50 mM Tris-HCl, pH 7.5, 500mM NaCl, and 5% glycerol. Finally, the protein fractions from the column were pooled, concentrated to the desired concentration, flash-frozen in liquid nitrogen, and stored at −80 °C for subsequent experiments.

### ThT fluorescence assay.

Aβ40 and Aβ42 (rPeptide, #A-1153-1, and #A-1163-2) with a purity of > 97%. Each peptide vial was suspended in 1,1,1,3,3,3-hexafluoro-2-propanol (HFIP), incubated for an hour to disperse any preexisting aggregates. Further these samples were aliquoted and carefully dried in nitrogen stream and stored in −80°C. Aliquots of purified Aβ species were dissolved in 10 mM NaOH to a concentration of 2 mg/ml followed by 10 min cooling and sonication in an ice water bath for 1 min. The concentration of the final monomeric sample was quantified by nanodrop at 280 nm. Purified MDK was dialyzed overnight in 50 mM Tris-HCl buffer, pH 7.5 and final concentration was determined by absorbance measurements at 280 nm Further, the freshly dissolved Aβ peptides were diluted to 5 µM in absence and presence of different concentrations of purified MDK containing 50 mM Tris-HCl, pH 7.5 buffer containing 20 µM Thioflavin. 100 µl of solution was added into 96-well half area, solid bottom, clear, sterile, microtiter plates and sealed with sealing tape to prevent evaporation. All kinetic experiments were performed at 37°C under quotient conditions at every 5 min in a Clariostar plate reader (BMG Labtech) using an excitation and emission wavelengths of 440 and 480 nm respectively. All these assays were performed in three replicates. Aggregation kinetics of MDK at different concentrations in the same buffer with fixed ThT concentrations were also recorded.

### NMR HSQC Spectroscopy.

The ^15^N-labeled Aβ40 and Aβ42 peptides (rPeptide, #A-1101-2 and #A-1102-2) were dissolved in 10mM NaOH to a concentration of 2 mg/ml, followed by 10 min cooling and 1 min sonication in an ice water bath. After aliquoting on ice, the samples were stored in a −80°C freezer. Subsequently, 10 µM of the ^15^N-labeled Aβ42 and Aβ40 peptides were incubated with 0, 5, and 10 µM MDK in low-binding tubes (Eppendorf, #0030108434) at 37°C for 24 and 48 h, respectively. The NMR spectra of all these samples including 10 µM monomeric Aβ species were acquired on Bruker Avance 600 MHz or 800 MHz at 278K. The spectrometers are equipped with a triple-resonance cryoprobe, and the data were processed in NMR Pipe^72^. and analyzed using NMRFAM-SPARKY^73^. The backbone resonance assignment of Aβ40 was taken from BMRB (ID: 17795). Binding of MDK to the 15N-labeled Aβ40 and Aβ42 was investigated using two-dimensional (2D) [^1^H-^15^N] so fast HMQC^74^ spectrum recorded either with 32 scans or 128 scans with an interscan delay of 0.2 s. The intensity of the peaks from different spectra were quantified, normalized with respect to the highest peak and used in analyzing the data.

### Circular dichroism spectroscopy.

Aβ aggregated triplicate samples in the absence and presence of MDK were collected directly from the 96-well plates. CD spectra of monomeric and fibrillated Aβ species (in the absence and presence of MDK) were recorded on a JASCO-1500 Spectrophotometer at a wavelength of 195 – 250 nm with a step size of 0.2 nm, 2 nm bandwidth, and a scan speed of 50 nm/min. CD spectra of MDK alone before and after incubation were also recorded. For each sample, the average of five scans was recorded and background correction was done by subtraction of corresponding buffer spectra. Spectra of native and aggregated MDK were collected with the same parameters at a wavelength of 200 to 250 nm.

### Negative stain transmission electron microscopy.

Aβ species alone and reactions involving Aβ together with 10 µM of MDK samples from the kinetic experiments were collected at the end point of the ThT experiments. 400-mesh copper grids (CF400-Cu grids, Electron Microscopy Sciences) were plasma cleaned with an Ar/O2 gas mixture for 10s using Solarus plasma cleaner (Gatan), followed by 5 µl of sample. The samples were allowed to adsorb for 1 min before blotting away the excess liquid, followed by rinsing using Milli Q water and subsequent staining using three successive applications of 2% uranyl acetate. The last round of stain application was allowed to sit for a min before blotting away the excess stain. The grids were air-dried prior to imaging using a 120 kV Talos L120C TEM (Thermo Fisher Scientific) equipped with a CETA detector (TFS).

### Immunogold negative-stain electron microscopy.

Extraction of Aβ filaments was performed as in the previous report^75^. Aβ filaments were deposited on glow-discharged 400 mesh formvar/carbon coated copper grids (EM Sciences CF400-Cu) for 40 s. Subsequently, the grids were blocked for 10 min with 1% BSA in PBS and incubated with anti-MDK (1:50, goat polyclonal, Bio-Techne, #AF-258-SP). After rinsing with blocking buffer, the grids were incubated with anti-goat IgG conjugated with 10 nm gold particle (1:20, Sigma), followed with wash and stained with 2% uranyl acetate for 1 min. Images were acquired at 11,000x with a Gatan Orius SC200B CCD detector on a Tecnai G2 Spirit at 120 kV.

### Detergent extraction for insoluble proteome.

The analysis procedure was adapted from a previously published method^19^. Mouse cortices were extracted with Lysis Buffer (50 mM HEPES, pH 7.5, 5 mM EDTA, 1 mM DTT, 1% Triton X-100, 0.5% sodium deoxycholate, 0.1% Sarkosyl, 10% glycerol, and 1x Sigma cOmplete Protease Inhibitor Cocktail). Protein lysates were centrifuged at 3000 x g for 5 min at 4°C. The resulting supernatant was subjected to a second centrifugation at 130,000 x g for 1 h at 4°C. The resulting pellet was washed with 50 mM HEPES containing 0.5% sodium deoxycholate and centrifuged at 17,200 x g for 30 min at 4°C. The insoluble pellet was subsequently resuspended in 8 M Urea with 0.5% sodium deoxycholate for proteome profiling.

### Mass spectrometry-based proteomics.

We used an optimized protocol of TMT-LC/LC-MS/MS for deep proteome profiling^44,45^. Protein samples were lysed by homogenization in the lysis buffer (50 mM HEPES, pH 8.5, 8 M urea, and 0.5% sodium deoxycholate), and their concentrations were measured by the BCA assay (Thermo Fisher Scientific, #23227) and confirmed by Coomassie-stained short SDS gels ^76^. Quantified protein samples (~0.1 mg per TMT channel) were digested with Lys-C (1:100 w/w, Wako, distributor, #121-05063) for 2 h at 21 °C, followed by dilution to decrease urea to 2 M and trypsin digestion (1:50 w/w, Promega, #V5113) overnight at 21 °C. Cys residues were reduced and alkylated by iodoacetamide. The proteolysis was terminated by adding trifluoroacetic acid to 1%. The resulting peptides were desalted with the Sep-Pak C18 cartridges (Waters), TMT-labeled (Thermo Fisher Scientific, #A34808 and A44520), and pooled equally. The pooled peptides were resolved by basic pH reverse phase LC on an XBridge C18 column (3.5 μm beads, 4.6 mm x 25 cm, Waters; buffer A: 10 mM ammonium formate, pH 8.0; buffer B: 95% acetonitrile, 10 mM ammonium formate, pH 8.0, ~2 h gradient, 40–80 concatenated fractions collected)^77^. Each fraction was analyzed by acidic pH LC-MS/MS (75 µm x ~20 cm, 1.9 µm C18 resin from Dr. Maisch GmbH, buffer A: 0.2% formic acid, 5% DMSO; buffer B: buffer A plus 65% acetonitrile, ~1.5 h gradient). The settings of Q Exactive HF Orbitrap MS (Thermo Fisher Scientific) included the MS1 scan (~410–1600 *m/z*, 60,000 resolution, 1 x 10^6^ AGC and 50 ms maximal ion time) and 20 data-dependent MS2 scans (fixed first mass of 120 *m/z*, 60,000 resolution, 1 x 10^5^ AGC, ~110 ms maximal ion time, HCD, 32–35% normalized collision energy, ~1.0 *m/z* isolation window with 0.3 *m/z* offset, and ~15 s dynamic exclusion)^45^.

The raw MS data were searched against protein database by the JUMP software (v1.13.4)^78^, which utilizes both pattern matching and *de novo* tag scoring to improve the sensitivity and specificity. A composite target/decoy database was used to evaluate FDR in peptide identification^79,80^. The protein target database combined downloaded Swiss-Prot, TrEMBL, and UCSC databases (human: 83,955 entries; mouse: 59,423 entries). Search parameters included precursor/product ion mass tolerance (± 10 ppm), full trypticity, static mass shift (TMT tags of 229.16293 and Cys carbamidomethylation of 57.02146 on cysteine), dynamic mass shift (Met oxidation of 15.99491), two maximal miscleavage sites, and three maximal modification sites. Peptide-spectrum matches (PSMs) were filtered by matching scores and mass accuracy to limit protein FDR below 1%.

Proteins were quantified from TMT reporter ions based on our published method^46^. Briefly, TMT reporter ion intensities were extracted for each PSM, corrected by isotopic distribution of TMT reagents, filtered to remove poor PSMs (e.g., minimum intensity of 1,000), and adjusted to alleviate sample pooling bias. The relative protein intensities were averaged from all assigned PSMs after removing outliers (e.g., Dixon’s Q-test or generalized extreme Studentized deviate test). Finally, the absolute protein intensities were derived by multiplying the relative intensities by the grand mean of top three abundant PSMs.

### Pathway enrichment by KEGG and gene ontology databases.

Pathway enrichment analysis was carried out by the JUMPn software (v1.13.0)^81^ to identify the biological functions of dysregulated genes/proteins in a given dataset. The analysis was performed using Fisher’s exact test against the Gene Ontology (GO) biological process, molecular function, and cellular component annotations, and KEGG pathway database, separately. The homologous genes between human and mouse were used as the background. The *p* values derived from Fisher’s exact test were further adjusted into FDR using the Benjamini-Hochberg procedure for multiple testing. Enriched pathways with FDR values < 0.05 were considered statistically significant.

### Protein-protein interaction (PPI) network analysis.

The analysis was performed based on our previously published protocol^82^ with modifications. DE genes/proteins were superimposed onto a composite PPI database by combining STRING (v11)^83^, BioPlex (v3.0)^84^, and InWeb_IM (v2016_09_12)^85^. The BioPlex database was developed by the method of affinity purification and mass spectrometry, whereas the STRING and InWeb contain information from various sources. Due to this heterogeneity of PPI interactions, the STRING and InWeb databases were further filtered by the edge score to ensure high quality, with the following rules: (i) only edges with evidence of physical interactions (e.g. through co-IP or yeast two-hybrid) were considered; (ii) edges of high confidence, as filtered by the edge score, with the cutoff determined by best fitting the log-log degree distribution using the scale free criteria^86^. The finally accepted STRING and InWeb databases were combined with BioPlex and the inhibitory postsynaptic density interactome^87^ to construct a composite PPI database, which includes 20,485 proteins and 1,152,607 PPI connections. PPI modules were then defined by a three-step procedure: (i) extracting a subnetwork by retaining PPI between two proteins if both were from the DE protein list; (ii) calculating a topologically overlapping measure (TOM)^88^ between each pair of proteins for the resulting PPI subnetwork, and (iii) dividing this network into individual modules based on the TOM clustering using the hybrid dynamic tree-cutting method^48^. The biological functions of each PPI module were further obtained using the proteins in each module as the input to perform the pathway enrichment analysis as described above.

### Quantitation and statistical analysis.

In small-scale analyses, two-tailed unpaired Student’s *t*-test was used for two-sample comparisons (Graphpad Prism 7.0.5). Proteomics analysis for differentially expressed proteins primarily utilized the limma R package (v3.48.3) ^89^ in several steps: (i) obtain the protein quantification data from MS as described above, (ii) perform a log transformation of the data^90^, (iii) calculate the *p* values by moderated *t*-test, and FDR values by the Benjamini-Hochberg procedure, using limma R package, (iv) calculate the mean for each protein under different conditions and derive the log2(fold change), (v) fit the log2(fold change) data of all proteins to a Gaussian distribution to generate a ‘global’ standard deviation value. Statistically significant changes were typically determined using an *P* value cutoff of 0.05 and a log2 (fold change) cutoff equivalent to two standard deviations.

## Supplementary Material

Supplement 1

## Data Availability

The mass spectrometry proteomics data were deposited to the ProteomeXchange Consortium via the PRIDE partner repository with the dataset identifiers PXD046539 (the whole proteome of AD mouse models), and PXD045746 (the aggregated proteome of the mice).
