# Supplementary material for: Midkine Attenuates Aβ Fibril Assembly and AmyloidPlaque Formation
Source: Res Sq. 2024 Jun 7:rs.3.rs-4361125. Preprint. [Version 1] doi: 10.21203/rs.3.rs-4361125/v1 (PMC11177971; doi:10.21203/rs.3.rs-4361125/v1)
Supplement: Supplement 1 [file NIHPPrs4361125v1-supplement-1.pdf]

Supplementary Files

This is a list of supplementary files associated with this preprint. Click to download.

- MDKExtendedDataTablesv1.0.0.xlsx
- ExtendedDataFig.docx
